# Digital self-management engagement and long-term patient-reported outcome trajectories in inflammatory arthritis: a real-world retrospective cohort study

**DOI:** 10.1007/s10067-026-08425-z

**Published:** 2026-09-29

**Authors:** Dmytro Fedkov, Danylo Yevstifeiev, Jan Leipe, Oleg Iaremenko, Daria Koliadenko, Liubov Petelytska, Felix Lang, Abdullah Khalil, Türker Kurt, Stefan Vordenbäumen

**Affiliations:** 1 Department of Internal Medicine, Bogomolets National Medical University, 13 Blvd Shevchenka, Kyiv, 01032 Ukraine; 2 Medical Center Medical Clinic Blagomed LLC, Kyiv, Ukraine; 3 Midaia GmbH, Heidelberg, Germany; 4 Rheumatology Department, Kiel Campus, Universitätsklinikum Schleswig-Holstein, Kiel, Germany; 5 Department of Rheumatology, St. Elisabeth-Hospital Meerbusch-Lank, Hauptstr. 74-76, 40668 Meerbusch, Germany; 6 Department of Rheumatology & Hiller Research Center, Medical Faculty, University Hospital Düsseldorf, Heinrich Heine University, Düsseldorf, Germany

**Keywords:** Arthritis, Psoriatic, Arthritis, Rheumatoid, Digital Health, Patient-reported outcome measures, Self-Management, Spondylarthropathies

## Abstract

**Introduction/objectives:**

Patient Global Assessment of Disease Activity (PtGADA), pain, and Routine Assessment of Patient Index Data 3 (RAPID3) are core patient-reported outcomes in inflammatory arthritis. We examined whether digital self-management engagement was associated with their long-term trajectories.

**Method:**

This retrospective cohort included 3513 adults using a certified digital self-management application. Patients were grouped by completion of 0, 1–2, or ≥3 structured self-management plans. Longitudinal models with patient-level cluster-robust standard errors estimated post-baseline change trajectories. Sensitivity analyses addressed differential attrition, measured confounding, and reverse causality.

**Results:**

Of 2895 patients with baseline PtGADA, 1026 (35.4%) contributed post-baseline observations. Patients completing ≥3 plans had more favourable PtGADA, pain, and RAPID3 trajectories than zero-plan patients (group-effect *p*=6.2×10^−5^, *p*=0.0055, and *p*=0.0044, respectively), without diverging slopes. At weeks 12–48, PtGADA improved by 11.1–12.1 points and pain by 7.2–7.3 points in the high-engagement group. After attrition weighting, PtGADA remained significant, RAPID3 retained a significant joint group effect, and pain was no longer significant.

**Conclusions:**

Higher engagement was most robustly associated with more favourable long-term PtGADA trajectories; support for pain was directionally similar but statistically more limited, and RAPID3 findings were the least consistent of the three outcomes. These results describe an engagement-associated phenotype rather than a causal treatment effect and support its evaluation as a potential marker of sustained digital engagement in future prospective stratification studies.

**Table:** 

**Key Points** • *In this real-world cohort of 3513 patients with inflammatory arthritis, higher digital self-management engagement was most robustly associated with more favourable PtGADA trajectories; the association with pain was directionally similar but less robust, while RAPID3 findings were less consistent.* • *Engagement-group trajectories were approximately parallel, and the findings remained directionally consistent in analyses examining sensitivity to differential follow-up, measured baseline differences, and reverse-causality concerns, although statistical significance varied by outcome and landmark.* • *The engagement-outcome association was directionally consistent across multiple sensitivity analyses addressing measured confounding and differential follow-up, although temporal exposure assignment could not be established and residual reverse-causality bias cannot be excluded.* • *Digital self-management engagement, operationalised by action-plan completion, represents a potential marker of sustained digital engagement for future prospective stratification studies, rather than a validated tool for baseline risk stratification.*

**Supplementary information:**

The online version contains supplementary material available at https://doi.org/10.1007/s10067-026-08425-z.

## Introduction

Modern management of inflammatory rheumatic and musculoskeletal diseases increasingly relies on outcomes reported directly by patients (patient-reported outcomes [PROs]) [1, 2]. Because pain and disease-related impact can fluctuate substantially between scheduled clinic visits, routine PRO monitoring captures important aspects of the patient’s day-to-day experience [3, 4]. Patient Global Assessment of Disease Activity (PtGADA) and pain assessed on a visual analogue scale (VAS) are recommended core outcome measures in rheumatoid arthritis (RA). They are also incorporated into treat-to-target recommendations and disease activity indices used in clinical trials and routine care [5, 6]. Routine Assessment of Patient Index Data 3 (RAPID3), a composite of function, pain, and patient global estimate scored on a 0–30 scale, has demonstrated utility for routine disease activity monitoring across inflammatory arthritis (IA) subtypes, including RA, psoriatic arthritis (PsA), and spondyloarthritis (SpA) [7–9].

Longitudinal patient global assessment trajectories in RA are persistent and heterogeneous. A landmark 15-year inception cohort study identified three stable trajectory phenotypes, with high patient global assessment scores explained more by pain and functional impairment than by objective inflammatory markers, underscoring the importance of the patient-reported experience [10, 11]. To address this multidimensional burden, the 2021 European Alliance of Associations for Rheumatology (EULAR) recommendations formally endorse structured self-management, goal-directed action planning, and digital healthcare as central elements of routine management [1].

Digital health platforms can operationalise these strategies while collecting longitudinal PROs data at scale in ecologically valid populations [12]. Despite their clinical utility, long-term PRO trajectories in real-world, digitally engaged populations remain poorly characterised. Most available evidence comes from randomised controlled trials or selected observational cohorts with follow-up limited to 12 to 24 weeks [13]. Meaningful differences may emerge over longer time horizons, and attending to heterogeneity in digital engagement intensity may reveal clinically important subgroups that are obscured in aggregate analyses.

The evaluation of long-term digital trajectories is methodologically challenging because sustained digital interventions often have high and non-random attrition. Evidence from adjacent contexts suggests that engagement intensity is associated with rapid outcomes: for example, mobile-collected RAPID3 data have shown clinically meaningful improvement as early as week 1 in patients initiating targeted therapies [14]. In the DiGAReal registry in Germany, which prospectively tracked prescribable digital health applications, 51% of users reported symptom improvement at 3 months, but no significant overall change in disease activity was detected, and only 15% completed the full program [15]. Similarly, a 12-month RA app study reported a mean of only 154 days until non-use and identified older age and more frequent physician engagement as predictors of continued use [16]. Across digital rheumatology studies, dropout rates frequently exceed 50%, making differential retention a critical contextual factor when interpreting long-term trajectory estimates and comparing highly engaged patients with those who disengage early [13, 17]. Understanding these digitally tracked trajectories while accounting for retention may inform future prospective stratification approaches [18].

To address the dual challenges of long-term tracking and engagement heterogeneity, we used data from the Mida Rheuma App (Midaia GmbH, Germany). This Conformité Européenne (CE)-certified Class IIa digital health application operationalises EULAR principles for adults with IA by enabling self-reported tracking of symptoms and functional status as well as engagement with structured self-management action plans across domains such as physical exercise, nutrition, and mindfulness. Previous evaluations of this platform have demonstrated short-term clinical utility, including improved disease activity control and dietary adherence in a 12-week randomised controlled trial [19, 20]. Real-world analyses have also highlighted the importance of patient phenotypes, showing that sustained adherence peaks in older patients with moderate-to-high baseline symptoms [21], and that psychosocial archetypes driven by sleep quality and fatigue predict 12-week pain relief and clinical remission [22, 23]. However, long-term outcome patterns associated with different levels of self-management intensity have not been well described.

Against this background, the central clinical question of the present study was whether patients who engage more actively with structured self-management action plans, operationalised by the number of fully completed plans, show more favourable longitudinal trajectories of PtGADA, pain, and RAPID3 over up to 114 weeks of follow-up. The study aimed to (1) characterise long-term PRO trajectories in a large real-world digital IA cohort; (2) examine whether action-plan completion intensity is associated with engagement-stratified trajectory differences across these three outcomes; (3) quantify the proportions of patients achieving clinically meaningful improvement at assessed time points by engagement group; and (4) assess the robustness of the primary findings through prespecified sensitivity analyses. Importantly, the study was not designed to evaluate treatment effectiveness; rather, it describes engagement-associated PRO trajectories and examines their potential clinical magnitude to inform future prospective evaluation of engagement-based stratification.

## Methods

### Study design and participants

We conducted a retrospective real-world cohort study using de-identified data from registered users of the Mida Rheuma App (Midaia GmbH, Germany), a CE-certified Class IIa medical device for adults with IA. Data were collected from the application’s launch in March 2021 through May 2026. The study complied with the Declaration of Helsinki (October 2024 revision) and Good Clinical Practice guidelines. The Local Ethics Committee of Medical Centre Medical Clinic Blagomed LLC approved the study protocol (protocol # 107/5, dated 13 Aug 2022). All patients provided digital informed consent prior to using the application. Reporting follows the Strengthening the Reporting of Observational Studies in Epidemiology (STROBE) guidelines for observational studies (Supplementary Table S1).

Eligible patients were 18 years of age or older and self-reported a physician-confirmed diagnosis of IA (RA, PsA, SpA, or mixed IA). Patients were required to have at least one valid baseline assessment for the analysed outcome, strictly defined as the first valid patient-recorded observation. For statistical adjustment, diagnosis strings were collapsed into a pragmatic phenotype: RA served as the reference category, alongside PsA, SpA (including axial and peripheral joint involvement), and mixed IA. Because this was a real-world observational study utilising all available cohort data within the specified timeframe, we did not perform a formal a priori sample-size calculation. Specific reasons for app non-use or loss to follow-up (e.g., lack of time, technical issues, or clinical remission) were not systematically captured by the application and were therefore unavailable for analysis.

The present analysis draws on the same underlying Mida Rheuma App patient population as these prior reports [21–23]; because the application’s user base and each patient’s available follow-up duration have continued to grow over time, the cohort analysed here is a larger and more extensively followed superset of those examined previously, rather than an independent sample.

### Exposure: action-plan completion intensity

The Mida Rheuma App provides up to 51 structured self-management action plans across evidence-based thematic domains, including exercise and movement, nutrition, sleep, mindfulness and mental health, pain management, and disease education [1, 24, 25]. Each plan contains a fixed number of tasks. We defined a plan as fully completed if the number of completed tasks equalled or exceeded the total assigned tasks, provided the total was greater than zero.

For each patient, we computed the total number of fully completed plans across all available slots. Patients were then categorised into three exposure groups: 0 completed plans, 1 to 2 completed plans, and 3 or more completed plans. We defined the threshold of 3 or more plans a priori to represent a high-engagement phenotype, reflecting sustained interaction across multiple domains. Conversely, the completion of 1 to 2 plans represented minimal or exploratory engagement. This exposure is time-invariant and is interpreted as an overall engagement phenotype rather than a time-ordered predictor.

### Study outcomes

The primary outcomes were patient-perceived disease impact and patient-reported pain. Disease impact was measured using PtGADA (0–100 electronic VAS) [26], and pain was measured using patient-reported pain (PPAIN; 0–100 electronic VAS) [27]. For both scales, higher scores indicate worse status. The secondary outcome was RAPID3 (scored 0–30), a validated composite of physical function, pain, and patient global estimate applicable across RA, PsA, and SpA [28–30]. We included RAPID3 due to its practical advantages for routine digital monitoring, while acknowledging that the index primarily captures patient-reported dimensions and may not fully reflect objective inflammatory signs.

For the primary longitudinal analysis, the dependent variable was the change from baseline, calculated as the current follow-up value minus the first valid observation. Clinically meaningful improvement was contextualised using established minimal clinically important difference (MCID) thresholds: a reduction of 10 points or greater for PtGADA and PPAIN [31, 32], and a reduction of 3.6 points or greater for RAPID3 [33, 34].

### Statistical analysis

The primary trajectory models were descriptive, estimating observed engagement-stratified outcome trajectories rather than causal treatment effects. Patients entered the trajectory cohort for each outcome if they had a valid baseline observation and at least one valid post-baseline observation within 114 weeks; patients with only a baseline observation and no further follow-up were not assigned an artificial zero-change value and did not contribute to the trajectory models, although their numbers are reported separately. Time was modelled in weeks since baseline.

We used linear regression models, chosen for parsimony and stability given the progressive sparsity of data at later time points. Cluster-robust standard errors at the patient level accounted for repeated measures. The models included engagement group and group-by-time interaction terms. Global tests for group terms and interactions used robust Wald tests aligned with the cluster-robust covariance framework. Because baseline observations were used to calculate change scores but were not included as model rows, the week-0 intercepts were extrapolated from post-baseline data and were not interpreted as observed baseline differences. Inference focused on model-derived estimates at weeks 12, 24, and 48, and on group-by-time interactions. For descriptive landmark responder analyses, the observation closest to each nominal landmark within a ±30-day window was used; the corresponding numbers of patients are reported in the tables. Missing continuous baseline covariates were imputed using the median and categorical covariates using the mode, where required; variable-specific missingness and imputation procedures are reported in Supplementary Table S2. Outcome values were not imputed.

### Sensitivity analyses

To assess the robustness of our findings and address potential confounding, we conducted several prespecified and exploratory sensitivity analyses. First, to reduce dependence on engagement accumulated over the entire follow-up period, we defined a restricted early-engagement proxy using only the first five action-plan slots and analysed observations from week 4 onward. Because exact action-plan completion timestamps were unavailable, this analysis did not establish strict temporal ordering and therefore could not eliminate immortal-time or reverse-causality bias. To further characterise the scale of this structural association between engagement and observation time, we compared account tenure (time from registration to last recorded login) across engagement groups. We also applied stabilised inverse probability weighting (IPW) to account for differential follow-up availability, defined as having valid outcome data at week 12 or later. The corresponding logistic model for follow-up availability included age, sex, diagnosis, baseline PtGADA, PPAIN, RAPID3, and action-plan group. Stabilised weights were upper-truncated at the 99th percentile to limit the influence of extreme values, and the resulting effective sample size was calculated using Kish’s formula to quantify the loss of statistical precision attributable to weight variability. To assess whether the small subset of zero-plans patients contributing post-baseline data was representative of the broader zero-plans population, we additionally compared baseline characteristics (age, sex, baseline PtGADA, and diagnosis distribution) between zero-plans patients who did and did not contribute post-baseline observations. In addition, multivariable-adjusted trajectory models incorporated age, sex, body mass index (BMI), diagnosis, and recorded baseline therapy class. The robustness of the exposure definition was further examined using alternative thresholds comparing patients who completed no plans with those who completed at least one plan and with those who completed three or more plans. To reduce the influence of sparse observations at later time points, the primary models were repeated after truncating follow-up at 60 and 80 weeks. Finally, patient-logged medications were mapped to six pharmacologic classes relevant to IA: conventional synthetic disease-modifying antirheumatic drugs (csDMARDs), biologic disease-modifying antirheumatic drugs (bDMARDs), targeted synthetic disease-modifying antirheumatic drugs including Janus kinase inhibitors (tsDMARDs/JAK inhibitors), glucocorticoids, nonsteroidal anti-inflammatory drugs (NSAIDs), and non-NSAID analgesics; baseline active records were compared across engagement groups. This analysis was intended only to assess recorded therapy exposure and not to provide a complete pharmacologic profile. We additionally tested diagnosis-by-engagement interaction terms to assess whether associations differed across RA, PsA, and SpA. As an additional exploratory analysis, Cox proportional hazards models were used with the early-engagement proxy to estimate time to the first outcome-specific MCID-defined improvement.

All statistical analyses were performed using R version 4.5.1 (R Foundation for Statistical Computing, Vienna, Austria). Two-sided *p* values <0.05 were considered statistically significant.

## Results

### Cohort characteristics

**Fig. 1:**
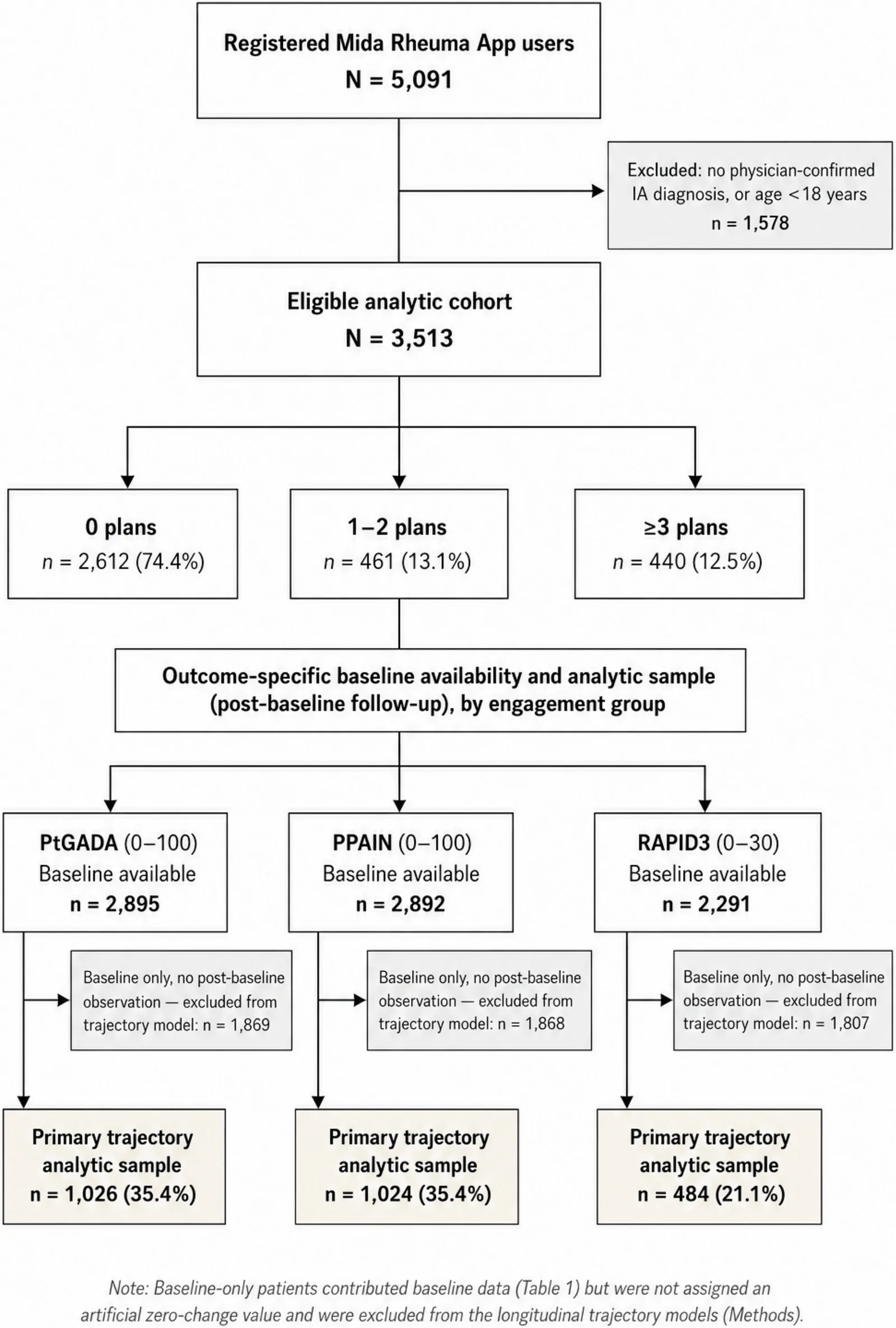
Strengthening the Reporting of Observational Studies in Epidemiology (STROBE) flow diagram of the analytic cohort and analysis samples. Baseline-only patients contributed baseline data but were not included in the longitudinal trajectory models. Abbreviations: IA, inflammatory arthritis; PPAIN, patient-reported pain; PtGADA, Patient Global Assessment of Disease Activity; RAPID3, Routine Assessment of Patient Index Data 3

Of 5091 registered app users, 3513 had a recorded eligible IA diagnosis and formed the primary analytic cohort (Fig. 1). Baseline PtGADA was available for 2895 patients, PPAIN for 2892, and RAPID3 for 2291. Regarding action-plan completion, 2612 patients (74.4%) completed zero plans, 461 (13.1%) completed 1–2 plans, and 440 (12.5%) completed ≥3 plans. Missingness in baseline covariates and the applied imputation methods are detailed in Supplementary Table S2.

The overall cohort was predominantly female (76.5%) with a mean age of 45.8 years (standard deviation [SD] 13.1). The diagnostic distribution comprised 55.8% RA, 13.9% PsA, 22.6% SpA, and 7.7% mixed IA. Baseline scores reflected moderate-to-severe disease impact: mean PtGADA was 58.3 (SD 25.5), PPAIN was 53.6 (SD 25.6), and RAPID3 was 12.8 (SD 5.3) (Table 1). Patients in the high-engagement group (≥3 plans) were on average 5.7 years older than those in the zero-plans group (50.2 vs. 44.5 years) and exhibited modestly lower baseline PtGADA (55.3 vs. 59.2) and PPAIN (49.8 vs. 54.7). This pattern aligns with previously described non-linear engagement behaviours in digital cohorts, where moderate symptom burden is associated with peak adherence [21].

**Table 1:** Baseline characteristics by action-plan completion group

Characteristic	0 plans (*n*=2612)	1–2 plans (*n*=461)	≥3 plans (*n*=440)	Overall (*N*=3513)
Age, mean (SD)	44.5 (13.1)	48.3 (12.3)	50.2 (12.2)	45.8 (13.1)
Female, n (%)	1959 (75.0%)	371 (80.5%)	356 (80.9%)	2686 (76.5%)
BMI, mean (SD)	27.9 (15.6)	27.2 (6.3)	26.6 (5.6)	27.6 (13.3)
Diagnosis				
RA, *n* (%)	1478 (56.6%)	232 (50.3%)	249 (56.6%)	1959 (55.8%)
PsA, *n* (%)	341 (13.1%)	88 (19.1%)	60 (13.6%)	489 (13.9%)
SpA, *n* (%)	584 (22.4%)	109 (23.6%)	100 (22.7%)	793 (22.6%)
Mixed IA, *n* (%)	209 (8.0%)	32 (6.9%)	31 (7.0%)	272 (7.7%)
Baseline PtGADA (0–100)				
Mean (SD)	59.2 (25.4)	56.9 (26.3)	55.3 (25.0)	58.3 (25.5)
*n* available	2050	434	411	2895
Baseline PPAIN (0–100 VAS)				
Mean (SD)	54.7 (25.6)	52.5 (25.9)	49.8 (25.0)	53.6 (25.6)
*n* available	2047	434	411	2892
Baseline RAPID3 (0–30)				
Mean (SD)	12.9 (5.3)	12.5 (5.4)	12.2 (5.2)	12.8 (5.3)
*n* available	1579	362	350	2291

Action-plan completion groups reflect the time-invariant engagement phenotype. Baseline outcome statistics are for patients with available baseline data for that specific outcome

*BMI* body mass index, *IA* inflammatory arthritis, *PPAIN* patient-reported pain assessed on a 0–100 visual analogue scale, *PsA* psoriatic arthritis, *PtGADA* Patient Global Assessment of Disease Activity (0–100 VAS), *RA* rheumatoid arthritis, *RAPID3* Routine Assessment of Patient Index Data 3 (0–30), *SD* standard deviation, *SpA* spondyloarthritis, *VAS* visual analogue scale

### Longitudinal trajectories by engagement group

In the primary continuous trajectory models, the pooled (non-engagement-stratified) cohort-level slope was not statistically significant for any outcome once baseline-only patients were excluded (PtGADA *p*=0.187; PPAIN *p*=0.559; RAPID3 *p*=0.930). Because a pooled slope combines a large, minimally engaged subgroup showing little change with a smaller, highly engaged subgroup showing more pronounced improvement, the cohort-average trend is difficult to interpret in isolation. Therefore, the engagement-stratified models below constitute our primary analysis.

**Fig. 2:**
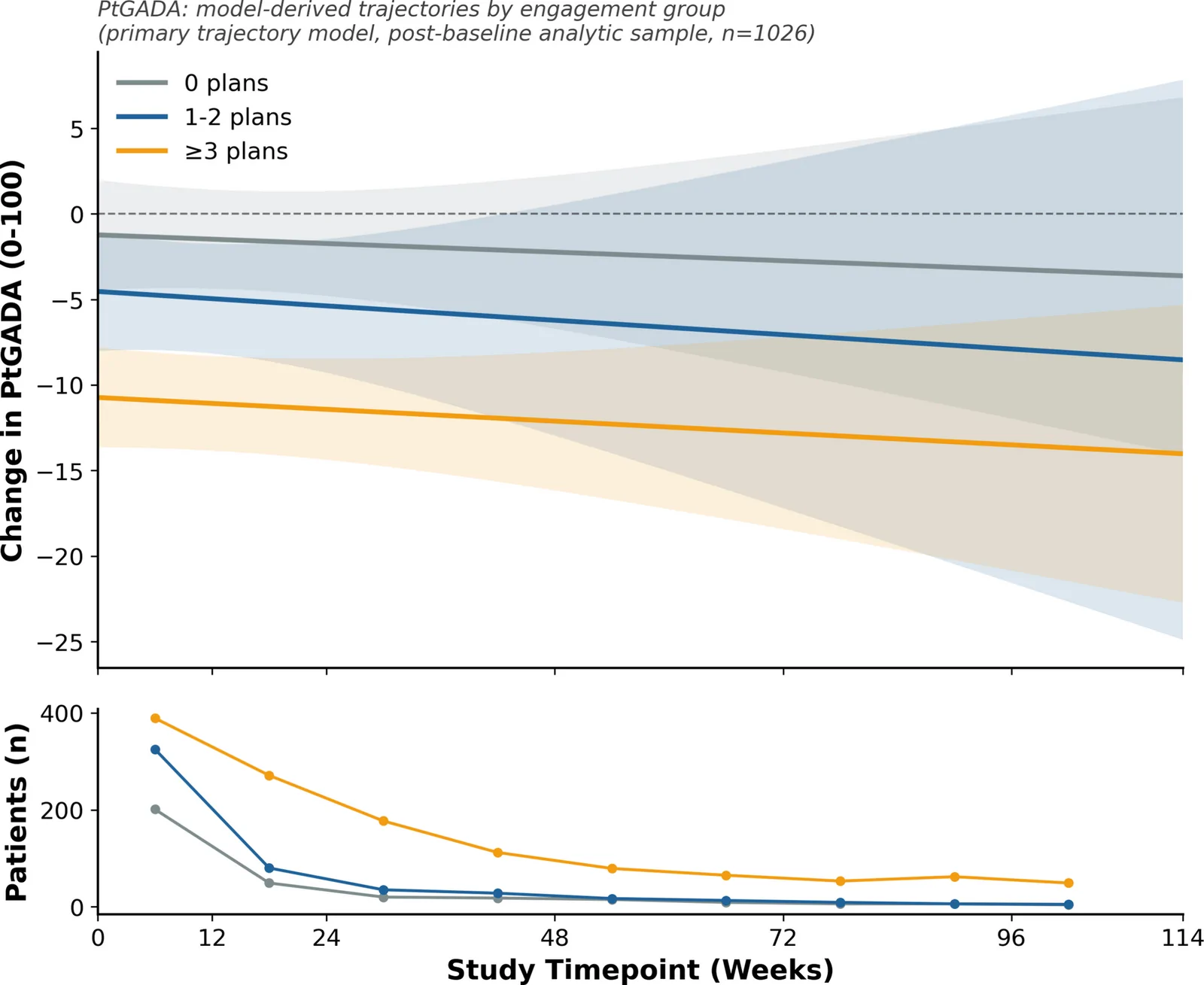
Longitudinal Patient Global Assessment of Disease Activity (PtGADA) trajectories according to digital self-management engagement. Model-derived mean change from baseline with 95% confidence intervals (CIs) and follow-up availability by engagement group are shown. Negative values indicate improvement. Because baseline observations were not included as model rows, estimates at or near week 0 are model extrapolations rather than observed baseline changes

**Fig. 3:**
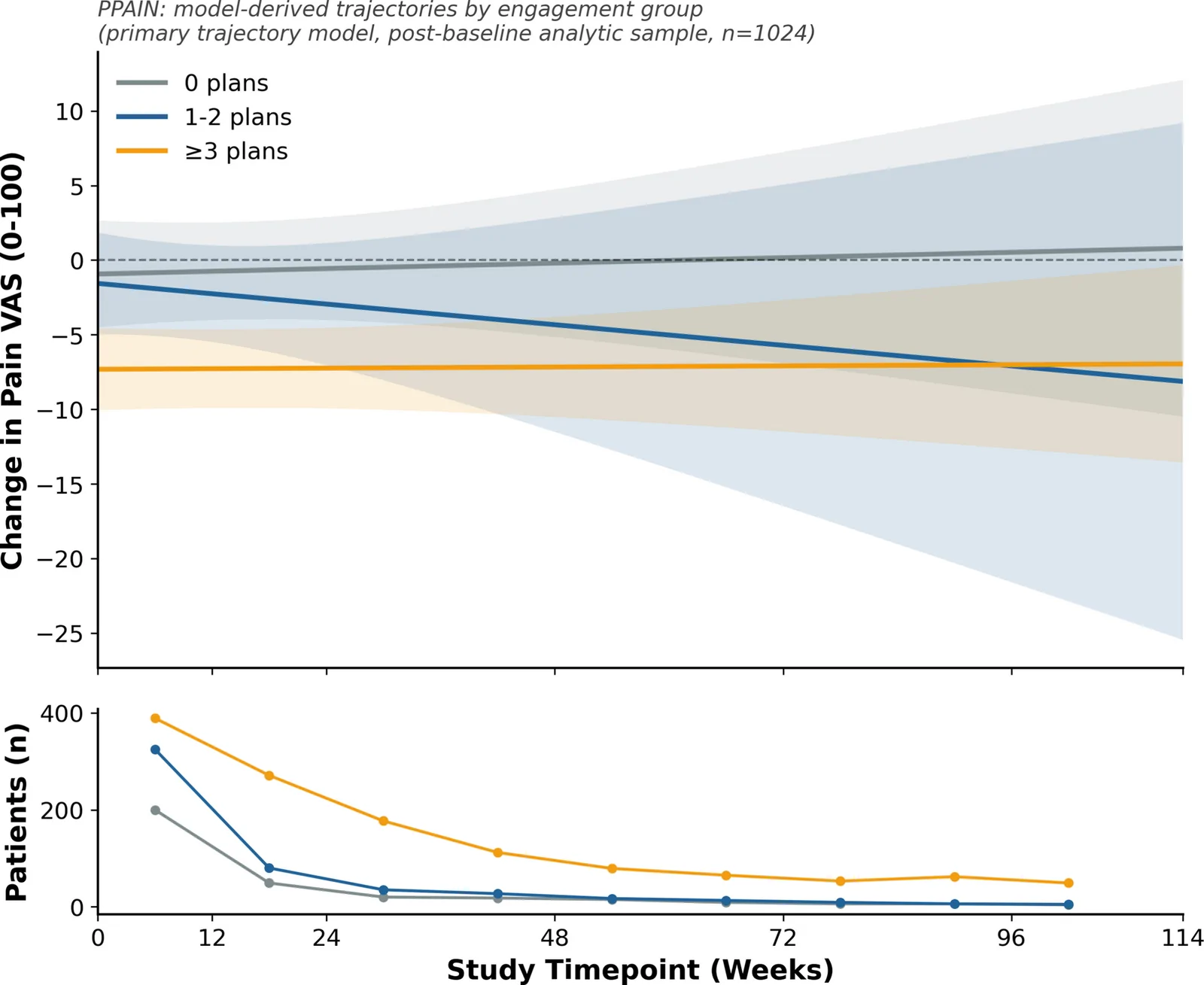
Longitudinal patient-reported pain (PPAIN) trajectories according to digital self management engagement. Model-derived mean change from baseline with 95% confidence intervals (CIs) and follow-up availability by engagement group are shown. Negative values indicate improvement. Because baseline observations were not included as model rows, estimates at or near week 0 are model extrapolations rather than observed baseline changes

Engagement-stratified models revealed a consistent association between action-plan completion intensity and favourable outcome trajectories (Figs. 2 and 3; Supplementary Figure S1). Robust Wald tests for the group main effect were significant across all outcomes (PtGADA *χ*^2^=19.37, *p*=6.2×10^−5^; PPAIN *χ*^2^=10.40, *p*=0.0055; RAPID3 *χ*^2^=10.83, *p*=0.0044). Because the week-0 intercepts were extrapolated from post-baseline data, these main effects were treated as omnibus indicators of group separation rather than as observed baseline differences. Group-by-time interaction terms were not statistically significant for any outcome (all *p*>0.05), providing no evidence of clearly diverging linear slopes. Model-derived time-point estimates with 95% confidence intervals (CIs) are detailed in Table 2.

**Table 2:** Model-derived change from baseline by action-plan completion group at assessed time points

Outcome, time point	0 plansmean (95% CI) *p*	1–2 plansmean (95% CI) *p*	1–2 minus 0 (*p*)	≥3 plansmean (95% CI) *p*	≥3 minus 0 (*p*)
A) PtGADA (0–100)—change from baseline					
Week 12	*n*=49 −1.48 (−4.37, +1.41) *p*=0.316	*n*=87 −4.95 (−8.16, −1.75) *p*=0.002	−3.48 *p*=0.115	*n*=303 −11.07 (−13.84, −8.31) *p*<0.001	−9.60 *p*<0.001
Week 24	*n*=27 −1.73 (−4.79, +1.33) *p*=0.268	*n*=41 −5.37 (−9.28, −1.47) *p*=0.007	−3.64 *p*=0.150	*n*=185 −11.42 (−14.37, −8.47) *p*<0.001	−9.69 *p*<0.001
Week 48	*n*=12 −2.23 (−6.71, +2.24) *p*=0.328	*n*=17 −6.22 (−12.95, +0.52) *p*=0.071	−3.98 *p*=0.335	*n*=79 −12.11 (−16.16, −8.07) *p*<0.001	−9.88 *p*=0.001
B) PPAIN (0–100)—change from baseline					
Week 12	*n*=49 −0.75 (−4.02, +2.52) *p*=0.654	*n*=87 −2.25 (−5.48, +0.98) *p*=0.172	−1.50 *p*=0.521	*n*=303 −7.27 (−9.90, −4.65) *p*<0.001	−6.53 *p*=0.002
Week 24	*n*=27 −0.57 (−4.02, +2.89) *p*=0.748	*n*=41 −2.94 (−7.03, +1.15) *p*=0.158	−2.38 *p*=0.384	*n*=185 −7.24 (−9.94, −4.53) *p*<0.001	−6.67 *p*=0.003
Week 48	*n*=12 −0.20 (−5.14, +4.73) *p*=0.936	*n*=17 −4.33 (−11.50, +2.85) *p*=0.237	−4.12 *p*=0.353	*n*=79 −7.16 (−10.52, −3.80) *p*<0.001	−6.96 *p*=0.022
C) RAPID3 (0–30)—change from baseline					
Week 12	*n*=71 −0.61 (−1.49, +0.27) *p*=0.172	*n*=60 +0.07 (−0.91, +1.05) *p*=0.891	+0.68 *p*=0.312	*n*=230 −1.60 (−2.12, −1.08) *p*<0.001	−0.99 *p*=0.056
Week 24	*n*=60 −0.60 (−1.36, +0.15) *p*=0.117	*n*=26 −0.32 (−1.21, +0.56) *p*=0.474	+0.28 *p*=0.639	*n*=153 −1.51 (−2.00, −1.01) *p*<0.001	−0.90 *p*=0.050
Week 48	*n*=16 −0.59 (−1.81, +0.64) *p*=0.347	*n*=11 −1.11 (−2.24, +0.03) *p*=0.055	−0.52 *p*=0.541	*n*=78 −1.32 (−1.96, −0.67) *p*<0.001	−0.73 *p*=0.303

Model-derived mean change from baseline (95% CI), within-group *p*-values, and between-group contrasts were obtained from the full longitudinal model using all eligible post-baseline observations. The *n* values indicate the number of patients with an observed assessment within ±30 days of the corresponding landmark and are provided only to show empirical data support at each time point. Negative values indicate improvement

*CI* confidence interval, *PPAIN* patient-reported pain assessed on a 0–100 visual analogue scale, *PtGADA* Patient Global Assessment of Disease Activity, *RAPID3* Routine Assessment of Patient Index Data 3, *VAS* visual analogue scale

**PtGADA:** The strongest engagement-related separation was observed for PtGADA. Among patients completing ≥3 plans, PtGADA was reduced by 11.07 points (95% CI −13.84 to −8.31; *p*<0.001) at week 12, 11.42 points (−14.37 to −8.47; *p*<0.001) at week 24, and 12.11 points (−16.16 to −8.07; *p*<0.001) at week 48. The zero-plans group showed non-significant changes from baseline at all time points, ranging from −1.48 to −2.23 points (*p*=0.27–0.33). Between-group differences (≥3 plans minus zero plans) were −9.60 points at week 12 (95% CI −13.60 to −5.59; *p*<0.001), −9.69 points at week 24 (95% CI −13.94 to −5.44; *p*<0.001), and −9.88 points at week 48 (95% CI −15.91 to −3.84; *p*=0.001). Patients completing 1–2 plans showed intermediate, non-significant differences versus the zero-plans group (*p*=0.11–0.33), consistent with a graded engagement-associated pattern.

**PPAIN:** Pain trajectories were broadly parallel to PtGADA. The high-engagement group experienced significant mean PPAIN reductions of 7.27 points (95% CI −9.90 to −4.65) at week 12, 7.24 points at week 24, and 7.16 points at week 48 (all *p*<0.001). Changes in the zero-plans group were small and non-significant. Between-group differences significantly favoured the high-engagement group at week 12 (−6.53 points; 95% CI −10.72 to −2.33; *p*=0.002), week 24 (−6.67 points; *p*=0.003), and week 48 (−6.96 points; *p*=0.022).

**RAPID3:** Trajectories were directionally consistent with PtGADA and pain. The high-engagement group achieved mean reductions of 1.60 points (95% CI −2.12 to −1.08; *p*<0.001) at week 12, 1.51 points at week 24, and 1.32 points at week 48 (all *p*<0.001). Between-group differences versus zero-completers approached but did not consistently reach conventional significance (week 12: −0.99 points, *p*=0.056; week 24: −0.90 points, *p*=0.050; week 48: −0.73 points, *p*=0.303). Given the lower availability of longitudinal RAPID3 data and its compressed scale range, these findings should be regarded as supportive rather than confirmatory.

### Clinically meaningful improvement

Analyses of clinically meaningful improvement (Supplementary Table S3) demonstrated a favourable response pattern in the high-engagement group. For PtGADA (≥10-point reduction), the proportion of responders was higher in the ≥3-plans group versus the zero-plans group at week 12 (49.8% vs. 38.8%; *p*=0.151), reaching significance at week 24 (43.2% vs. 18.5%; *p*=0.014) and week 48 (45.6% vs. 0.0%; *p*=0.003). Individual-level PtGADA responses at week 12 are shown in the waterfall plot in Supplementary Figure S2. For PPAIN, the high-engagement group showed significantly more responders at week 12 (44.6% vs. 28.6%; *p*=0.036) and week 48 (43.0% vs. 8.3%; *p*=0.025). For RAPID3, the clearest separation occurred at week 24 (41.8% vs. 13.3%; *p*<0.001). We note that responder proportions at late landmarks rely on small samples in the lower-engagement groups, and individual time-point comparisons should be interpreted with caution.

### Follow-up density

 Follow-up availability differed markedly across engagement groups (Table 3; Supplementary Table S4). In the zero-plans group, only 13.0% of patients with valid baseline data contributed any post-baseline PtGADA observation, and only 2.4% (49 of 2050) provided an observation within the week-12 landmark window; just 0.6% did so within the week-48 window. In contrast, 98.8% of patients completing ≥3 plans contributed post-baseline data, with 73.7% falling within the week-12 window and 19.2% within the week-48 window. The median (interquartile range [IQR]) number of post-baseline observations per followed patient was 1 (1–2) in the zero-plans group versus 9 (5–19) in the ≥3-plans group. The extreme differential retention implies that landmark estimates in the lower-engagement groups, especially at later time points, are based on a highly selected minority, representing a key limitation for between-group comparisons.

**Table 3:** Follow-up density: patients contributing per time bin, by action-plan completion group

Time bin (weeks)	0 plans, *n*	1–2 plans, *n*	≥3 plans, *n*	Total, *n*
PtGADA (0–100)				
Baseline (week 0)	2050	434	411	2895
Weeks 1–12	187	312	388	887
Weeks 13–24	43	69	238	350
Weeks 25–36	18	31	161	210
Weeks 37–48	15	23	108	146
Weeks 49–60	11	12	71	94
Weeks 61–72	7	13	61	81
Weeks 73–84	6	9	50	65
Weeks 85–96	4	5	58	67
Weeks 97–114	4	8	54	66
PPAIN (0–100 VAS)				
Baseline (week 0)	2047	434	411	2892
Weeks 1–12	186	312	388	886
Weeks 13–24	43	69	238	350
Weeks 25–36	18	31	161	210
Weeks 37–48	15	22	108	145
Weeks 49–60	11	12	71	94
Weeks 61–72	7	13	61	81
Weeks 73–84	6	9	50	65
Weeks 85–96	4	5	58	67
Weeks 97–114	4	8	54	66

*n* denotes patients contributing at least one valid observation within the time bin. PtGADA and PPAIN were captured concurrently.

*PPAIN* patient-reported pain assessed on a visual analogue scale, *PtGADA* Patient Global Assessment of Disease Activity, *VAS* visual analogue scale

### Sensitivity analyses

Selected primary and sensitivity results are summarised in Supplementary Table S5; additional prespecified and exploratory analyses are reported as follows:

- **Early-engagement proxy:** Using only the first five plan slots for observations from week 4 onward preserved the direction and magnitude of the primary finding for both PtGADA (high-engagement group, −11.54 points; *p*<0.001) and PPAIN (−7.75 points; p=0.004), providing partial reassurance against, but not excluding, reverse causation.
- **Observation weighting:** Under IPW, the high-engagement coefficient remained significant for PtGADA (−7.67 points, *p*=0.001), whereas the PPAIN coefficient was attenuated and no longer statistically significant (−4.02 points, *p*=0.143). In joint weighted models, the group effect remained significant for RAPID3 (*p*=0.015) but not for PPAIN; PtGADA also retained a significant high-engagement coefficient. For the PtGADA follow-up model, stabilised weights before truncation ranged from 0.21 to 6.30, with a 99th percentile of 5.13, among the followed-up subsample (*n*=532). Weights above the 99th percentile were upper-truncated at 5.13. The resulting effective sample size was approximately 192, corresponding to 36% of the nominal follow-up sample and indicating substantial variability in the inverse probability weights. Interaction analyses revealed no significant effect modification by underlying diagnosis for any outcome (all Wald test *p*>0.4), indicating consistent associations across RA, PsA, and SpA subgroups. Zero-plan subgroup representativeness: Among zero-plan patients with baseline PtGADA, those who did (*n*=266) and did not (*n*=1784) contribute post-baseline observations did not differ significantly in age, sex, baseline PtGADA, or diagnosis distribution (all *p*≥0.15).
- **Multivariable adjustment:** The high-engagement association remained statistically significant after adjustment for available recorded baseline variables, including age, sex, diagnosis, BMI, and recorded baseline therapy classes (group main effect: PtGADA *p*=0.0014, PPAIN *p*=0.012, RAPID3 *p*=0.006). Because medication data were voluntarily self-logged, these adjusted models should be interpreted as sensitivity analyses rather than definitive control for pharmacological confounding.
- **Alternative exposure thresholds:** Engagement of any degree (≥1 plan) was associated with significantly more favourable PtGADA trajectories relative to zero plans (−8.50 points; *p*<0.001), consistent with a graded rather than strict threshold pattern.
- **Time-window truncation:** Truncation at 60 and 80 weeks did not materially change the direction of the engagement-associated trajectory patterns.
- **Therapy balance:** Recorded baseline medication use was higher in the ≥3-plans group across all examined pharmacologic classes (e.g., csDMARDs: 18.7% vs. 9.9% in the zero-plans group, *p*<0.001; Supplementary Table S6). Because medication data were voluntarily self-logged, this pattern may reflect greater overall engagement with the app’s logging features rather than an isolated difference in treatment intensity.
- **Exploratory Cox analysis:** A Cox proportional-hazards model using the early-engagement proxy yielded hazard ratios of 3.10 (95% CI 2.35–4.08; *p*<0.005) for PtGADA, 2.61 (95% CI 1.96–3.48; *p*<0.005) for PPAIN, and 1.74 (95% CI 1.18–2.56; *p*<0.005) for RAPID3. Because exact action-plan completion timestamps were unavailable, temporal exposure ordering could not be established; these estimates should therefore not be interpreted as evidence of faster improvement and do not independently validate the primary trajectory findings. They are reported solely as hypothesis-generating.

## Discussion

In this real-world digital cohort of patients with IA, higher digital self-management engagement was associated with more favourable long-term trajectories of patient-reported disease impact and pain, with supportive but less consistent findings for RAPID3. The most robust and clinically interpretable signal was observed for PtGADA, with closely parallel patterns for pain. Patients in the high-engagement group (≥3 completed plans) showed sustained model-derived reductions in disease impact, with a substantial proportion also meeting the threshold for clinically meaningful pain improvement (Supplementary Table S3), although the group-level mean pain reduction did not itself reach the 10-point MCID threshold. Conversely, patients who completed zero plans showed no significant departure from their baseline state. However, because engagement was defined cumulatively rather than assigned randomly, these findings describe an engagement-associated phenotype rather than a confirmed causal treatment effect.

The approximately parallel trajectories across engagement groups, with separation evident at post-baseline landmarks rather than through progressively diverging slopes, indicate that between-group differences were already apparent at early post-baseline landmarks and remained broadly maintained thereafter. This observation, supported by the lack of significant time-by-engagement interactions, aligns with findings from the CareRA trial, which demonstrated that early and persistent treatment responses drive long-term self-efficacy in early RA more strongly than cumulative treatment duration [35]. It is also consistent with broader digital health patterns, where app-based interventions often show their largest observable changes during an early engagement window, followed by trajectories that remain relatively stable thereafter [36, 37]. Importantly, this pattern remained consistent across RA, PsA, and SpA subgroups, supporting the cross-diagnostic relevance of the engagement-outcome association [38].

The strong parallelism between PtGADA and pain trajectories reflects established clinical observations that high patient global assessments are driven largely by pain and functional limitations rather than systemic inflammation alone [10, 39]. While severe acute symptom flares might intuitively motivate patients to seek digital support, acute distress can also induce “digital fatigue” or reduce the cognitive bandwidth required to complete structured self-management tasks [40, 41]. The PtGADA association remained statistically significant after multivariable adjustment and inverse-probability weighting; however, residual confounding and selection related to differential follow-up cannot be excluded.

For the secondary composite outcome, RAPID3, between-group separation was more modest in absolute terms, consistent with its narrower 0–30 scale and its primary sensitivity to subjective patient-reported dimensions rather than objective systemic inflammation [42]. As with pain, the group-level mean RAPID3 reduction in the high-engagement group (1.3–1.6 points across landmarks) did not reach the pre-specified 3.6-point MCID threshold, so PtGADA, pain, and RAPID3 findings should not be regarded as equally robust. Despite this, individual-level analyses highlighted substantial complementary value: at week 24, the proportion of highly engaged patients achieving a clinically meaningful RAPID3 improvement was more than three times higher than that of the zero-plans group.

Several mechanisms may plausibly underlie the association between action-plan completion and more favourable PROs. The Mida Rheuma App incorporates evidence-based self-management domains recommended by 2021 EULAR guidelines [1], including physical exercise [25], anti-inflammatory nutrition [43], mindfulness [44, 45], and pain coping. In IA, non-pharmacological interventions targeting these areas are known to improve function and reduce pain [24]. Patient self-efficacy, the confidence in one’s ability to manage disease, may be one relevant mediator. Favourable early symptom improvement may contribute to greater self-efficacy, which has been associated with better subsequent functional outcomes.

A critical contextual factor in interpreting these results is the steep attrition rate, typical of real-world digital therapeutics. While the highly engaged group maintained robust follow-up, the zero-plans group exhibited extreme dropout, aligning with reviews of decentralised clinical trials where substantial proportions of participants abandon interventions almost immediately [17, 36]. Comparison with other digital IA registries further highlights this challenge: The DiGAReal registry reported high attrition and no significant overall change in disease activity at 3 months [15]. Our study extends this evidence base by showing that a subset of patients who sustained high digital engagement experienced substantial long-term symptomatic improvement, consistent with prior evidence that older age and early task completion are predictors of digital adherence in RA [16].

### Strengths and limitations

The principal strengths of this study include its large real-world sample size, extended follow-up duration, and a multi-outcome design capturing both global impact (PtGADA) and pain. By restricting the primary trajectory models to patients with genuine post-baseline observations, we avoided the distortion introduced by artificial zero-change rows for baseline-only patients. Furthermore, sensitivity analyses including the restricted early-engagement proxy, IPW, multivariable adjustment, alternative exposure thresholds, and follow-up truncation examined the robustness of the findings to several key methodological challenges of real-world digital health studies, although residual bias could not be excluded.

Several limitations should be acknowledged. Because completing three or more plans requires remaining actively engaged with the application for a sustained period, the high-engagement group is structurally composed of patients who, by definition, remained under observation longer—an immortal-time-bias concern that affects both the primary trajectory models and the exploratory Cox analysis. Consistent with this, median (IQR) account tenure (time from registration to last recorded login) was 11.0 (0.9–41.5) weeks in the zero-plans group versus 65.7 (33.6–124.4) weeks in the ≥3-plans group, illustrating the scale of this structural imbalance. A landmark analysis restricting exposure ascertainment to a fixed early time point was not feasible with the available data due to the absence of exact action-plan completion timestamps, which also precludes strict causal inference. Furthermore, the time-invariant exposure classification prevents modelling within-patient fluctuations in engagement over time.

Differential retention was substantial across engagement groups. Although this issue was evaluated using IPW, landmark estimates for the zero-plans group were necessarily based on a highly selected minority of the baseline sample. Although zero-plan patients with and without post-baseline observations did not differ materially in the measured baseline characteristics, selection on unmeasured factors cannot be excluded. Under IPW, the single-coefficient engagement effect for PPAIN was attenuated to non-significance (*p*=0.143), consistent with the joint Wald test for the full weighted model (*p*=0.31; Sensitivity Analyses), suggesting that the pain signal was particularly sensitive to the missing-data mechanism and selective dropout. Given the pronounced differences in follow-up availability across engagement groups, limited empirical positivity and residual instability of the stabilised weights cannot be excluded. IPW diagnostics indicated substantial weight variability and reduced effective sample size, consistent with limited empirical positivity.

Residual confounding by indication and the healthy adherer effect also remain possible. Patients may modify their app use in response to symptom severity or anticipated relief, while highly engaged users may have stronger health-seeking behaviours and better adherence to background disease-modifying pharmacotherapy.

Medication data were incomplete because only 31.0% of patients voluntarily recorded their treatments. Although baseline medication recording was more frequent in the engaged group across all drug classes, it was not possible to determine whether this reflected true differences in pharmacological intensity or simply a greater tendency to use the app’s logging functions. More broadly, objective markers of inflammatory disease activity (C-reactive protein, erythrocyte sedimentation rate, or swollen/tender joint counts) were not collected by the application at any time point, so the study characterises engagement-associated patterns in patient-reported outcomes only, without corresponding evidence on objective disease activity. Diagnoses were likewise self-reported by patients rather than independently verified against medical records or clinician-entered data, so some misclassification between IA subtypes is possible and could not be quantified. Residual confounding by these unmeasured or unverified factors therefore cannot be excluded.

Finally, the cohort consisted predominantly of female, digitally engaged patients from German-speaking settings, which may limit generalisability to broader, less digitally literate, or more demographically diverse populations.

## Conclusion

In this real-world digital IA cohort with follow-up extending to 114 weeks, higher digital self-management engagement, operationalised as completion of structured self-management plans, was most consistently and robustly associated with more favourable PtGADA trajectories; support for pain improvement was directionally concordant but statistically more limited, and findings for RAPID3 were the least consistent of the three outcomes. These associations showed a graded engagement pattern and remained directionally consistent in analyses examining sensitivity to differential follow-up, measured baseline differences, and reverse-causality concerns, although statistical significance varied across individual landmarks and outcomes. Because engagement was not randomised and was defined cumulatively, the findings should be interpreted as an engagement-associated phenotype, rather than as evidence of causal treatment effectiveness. The results support prospective evaluation of action-plan completion intensity as a potential marker of sustained digital engagement in future stratification studies.

## Supplementary information

Below is the link to the electronic supplementary material.

ESM 1 (DOCX 451 KB)

## Data Availability

Midaia GmbH maintained the source application database. All authors, including the non-company academic co-authors, had access to the underlying de-identified patient-level dataset under the applicable data-processing arrangements. Formal statistical analyses were performed independently by the designated study investigators, and the authors retained full control over the reported analyses, interpretation of the findings, and manuscript content. The underlying dataset is not publicly available because it is proprietary and subject to applicable data protection agreements. However, de-identified data required for confidential editorial or peer-review verification may be made available to the journal upon reasonable request, subject to applicable data protection requirements.
