# Supplementary material for: Digital self-management engagement and long-term patient-reported outcome trajectories in inflammatory arthritis: a real-world retrospective cohort study
Source: Clin Rheumatol. 2026 Sep 29;45(11):6999–7013. doi: 10.1007/s10067-026-08425-z (PMC13723565; doi:10.1007/s10067-026-08425-z)
Supplement: Supplementary file 1 [file 10067_2026_8425_MOESM1_ESM.docx]

**SUPPLEMENTARY MATERIAL**

**Supplementary Table S1.** **STROBE Statement—checklist of items that should be included in reports of observational studies**

|  | | Item No. | Recommendation | Relevant text from manuscript | |  | |
| --- | --- | --- | --- | --- | --- | --- | --- |
| **Title and abstract** | | 1 | (a) Indicate the study’s design with a commonly used term in the title or the abstract | **Title**: "...a real-world retrospective cohort study" | |  | |
|  |  |  | (b) Provide in the abstract an informative and balanced summary of what was done and what was found | **Abstract (Method): "This retrospective cohort study included..."** | |  | |
| Introduction | | | |  | |  | |
| Background/rationale | | 2 | Explain the scientific background and rationale for the investigation being reported | **Introduction**: Paragraphs 1-5 (rationale on PRO monitoring, digital interventions, high attrition, and the Mida Rheuma App platform). | |  | |
| Objectives | | 3 | State specific objectives, including any prespecified hypotheses | **Introduction**: Paragraph 6 (“The study aimed to: (1) characterize... (2) examine... (3) quantify... (4) assess...”). | |  | |
| Methods | | | |  | |  | |
| Study design | | 4 | Present key elements of study design early in the paper | **Methods (Study Design and Participants)**: Paragraph 1. | |  | |
| Setting | | 5 | Describe the setting, locations, and relevant dates, including periods of recruitment, exposure, follow-up, and data collection | **Methods (Study Design and Participants)**: "...from the application's launch in March 2021 through May 2026." | |  | |
| Participants | | 6 | (a) Cohort study—Give the eligibility criteria and the sources and methods of selection of participants. Describe methods of follow-up  Case-control study—Give the eligibility criteria and the sources and methods of case ascertainment and control selection. Give the rationale for the choice of cases and controls  Cross-sectional study—Give the eligibility criteria and the sources and methods of selection of participants | **Methods (Study Design and Participants)**: "Eligible patients were 18 years of age or older; had a self-reported physician-confirmed inflammatory arthritis diagnosis... and had at least one valid baseline assessment..." | |  | |
|  |  |  | (b) Cohort study—For matched studies, give matching criteria and number of exposed and unexposed  Case-control study—For matched studies, give matching criteria and the number of controls per case | N/A (Not a matched cohort study). | |  | |
| Variables | | 7 | Clearly define all outcomes, exposures, predictors, potential confounders, and effect modifiers. Give diagnostic criteria, if applicable | **Methods**: Sections "Exposure: Action-Plan Completion Intensity" and "Outcomes" (PtGADA, PPAIN, RAPID3). | |  | |
| Data sources/ measurement | | 8* | For each variable of interest, give sources of data and details of methods of assessment (measurement). Describe the comparability of assessment methods if there is more than one group | **Methods**: All data were sourced from the CE-certified Mida Rheuma App. Outcomes measured via a 0–100 electronic VAS and the RAPID3 index. | |  | |
| Bias | | 9 | Describe any efforts to address potential sources of bias | **Methods (Sensitivity Analyses):** IPW was used to address differential follow-up; a restricted early-engagement proxy and account-tenure analysis were used to characterise the structural association between engagement and observation time, although immortal-time and reverse-causality bias could not be eliminated. | |  | |
| Study size | | 10 | Explain how the study size was arrived at | **Methods (Study Design and Participants)**: "Because this was a real-world observational study using all available cohort data... no formal a priori sample size calculation was performed." | |  | |
| Quantitative variables | | 11 | Explain how quantitative variables were handled in the analyses. If applicable, describe which groupings were chosen and why | | **Methods (Exposure)**: Continuous plans categorized into 0, 1–2, and ≥3 groups (threshold chosen a priori to represent 'high engagement'). | |  |
| Statistical methods | | 12 | (a) Describe all statistical methods, including those used to control for confounding | | **Methods (Statistical Analysis)**: Continuous trajectory models with cluster-robust standard errors, IPW models including covariates (age, sex, diagnosis, baselines). | |  |
|  |  |  | (b) Describe any methods used to examine subgroups and interactions | | **Methods (Statistical Analysis)**: "Group and group-by-time interaction terms were included... an interaction analysis was conducted by adding a diagnosis-by-engagement interaction term..." | |  |
|  |  |  | (c) Explain how missing data were addressed | | Methods (Statistical Analysis): missing continuous baseline covariates were imputed using the median and categorical covariates using the mode, where required; variable-specific missingness is reported in Supplementary Table S2. Outcome missingness was evaluated through the longitudinal analysis structure and IPW sensitivity analyses. | |  |
|  |  |  | (d) Cohort study—If applicable, explain how loss to follow-up was addressed  Case-control study—If applicable, explain how matching of cases and controls was addressed  Cross-sectional study—If applicable, describe analytical methods taking account of sampling strategy | | **Methods (Statistical Analysis)**: Stabilized inverse probability weighting (IPW) was applied for differential follow-up availability. | |  |
|  |  |  | (e) Describe any sensitivity analyses | | **Methods (Statistical Analysis)**: Multiple prespecified and exploratory sensitivity analyses, including an early-engagement proxy, observation weighting/IPW, comparison of zero-plan patients with and without post-baseline follow-up, multivariable adjustment, alternative exposure thresholds, time-window truncation, therapy-balance assessment, diagnosis-by-engagement interaction testing, and exploratory time-to-event analysis. | |  |
| Results | | | | | | |  |
| Participants | | 13* | (a) Report numbers of individuals at each stage of study—eg numbers potentially eligible, examined for eligibility, confirmed eligible, included in the study, completing follow-up, and analyzed | | **Results (Cohort Characteristics)**: "Of 5,091 registered app users, 3,513 had a recorded eligible... diagnosis and formed the primary analytic cohort." See also Figure 1. | |  |
|  |  |  | (b) Give reasons for non-participation at each stage | | **Methods (Study Design and Participants)**: "...specific reasons for app non-use or loss to follow-up... were not systematically captured by the application..." | |  |
|  |  |  | (c) Consider the use of a flow diagram | | **Figures**: Figure 1 (STROBE flow diagram). | |  |
| Descriptive data | | 14* | (a) Give characteristics of study participants (eg demographic, clinical, social) and information on exposures and potential confounders | | **Results (Cohort Characteristics)** & **Table 1**. | |  |
|  |  |  | (b) Indicate the number of participants with missing data for each variable of interest | | **Table 1**: "n available" row for Baseline PtGADA, PPAIN, and RAPID3. | |  |
|  |  |  | (c) Cohort study—Summarise follow-up time (eg, average and total amount) | | **Results** (Follow-up Density), Table 3, and Supplementary Table S4 report outcome availability across 12-week intervals up to 114 weeks; the median number of post-baseline observations per followed patient is also reported in Results. | |  |
| Outcome data | | 15* | Cohort study—Report numbers of outcome events or summary measures over time | | **Results**: Table 2 (model-derived change from baseline at landmarks) and Table 3 (number of participants per time bin). | |  |
| Main results | | 16 | (a) Give unadjusted estimates and, if applicable, confounder-adjusted estimates and their precision (eg, 95% confidence interval). Make clear which confounders were adjusted for and why they were included | | **Results**: Table 2 (mean differences and 95% CIs) & "Sensitivity Analyses" section (IPW-adjusted trajectory estimates). | |  |
|  |  |  | (b) Report category boundaries when continuous variables were categorized | | **Table 2**: 0 plans, 1–2 plans, and ≥3 plans. | |  |
|  |  |  | (c) If relevant, consider translating estimates of relative risk into absolute risk for a meaningful time period | | Not applicable because relative risks were not estimated; absolute responder proportions by engagement group and time point are reported in Supplementary Table S3. | |  |
| Other analyses | | 17 | Report other analyses done—eg analyses of subgroups and interactions, and sensitivity analyses | | **Results (Sensitivity Analyses)** & **Supplementary Table S5**. | | |
| Discussion | | | | | | | |
| Key results | 18 | | Summarise key results with reference to study objectives | | **Discussion:** Paragraph 1 (higher digital self-management engagement was associated with more favorable long-term patient-reported outcome trajectories). | | |
| Limitations | 19 | | Discuss limitations of the study, taking into account sources of potential bias or imprecision. Discuss both the direction and the magnitude of any potential bias | | **Discussion (Strengths and Limitations)**: Key limitations include the cumulative exposure definition and associated immortal-time bias, extreme differential retention, residual confounding and the healthy-adherer effect, incomplete self-recorded medication data, absence of objective inflammatory markers, potential diagnostic misclassification, and limited generalisability. | | |
| Interpretation | 20 | | Give a cautious overall interpretation of results considering objectives, limitations, multiplicity of analyses, results from similar studies, and other relevant evidence | | **Discussion:** Paragraphs 2-6 (Contextualized with CareRA trial, DiGAReal registry) & Conclusion: "...should be interpreted as engagement-associated trajectories rather than evidence of causal treatment effectiveness." | | |
| Generalisability | 21 | | Discuss the generalisability (external validity) of the study results | | **Discussion (Strengths and Limitations)**: "...possible selection bias towards digitally engaged, predominantly female patients within German-speaking settings, which may limit the broader generalisability..." | | |
| Other information | | |  | | | | |
| Funding | 22 | | Give the source of funding and the role of the funders for the present study and, if applicable, for the original study on which the present article is based | | **Funding/role of sponsor**: "This work was supported by a European Alliance of Associations for Rheumatology (EULAR) Research Grant... The funding body had no role in study design, analysis, interpretation, or manuscript preparation." | | |

Abbreviations: CE, Conformité Européenne; EULAR, European Alliance of Associations for Rheumatology; IA, inflammatory arthritis; IPW, inverse probability weighting; N/A, not applicable; PPAIN, patient-reported pain; PRO, patient-reported outcome; PtGADA, Patient Global Assessment of Disease Activity; RAPID3, Routine Assessment of Patient Index Data 3; STROBE, Strengthening the Reporting of Observational Studies in Epidemiology; VAS, visual analogue scale.

**Supplementary Table S2. Missingness and imputation for covariates used in IPW and adjusted models**

| **Variable** | **Available, n (%)** | **Missing, n (%)** | **Imputation method** |
| --- | --- | --- | --- |
| Age | 3,494 (99.5%) | 19 (0.5%) | Median imputation |
| Sex | 3,484 (99.2%) | 29 (0.8%) | Mode imputation |
| Diagnosis (RA/PsA/SpA/Mixed) | 3,513 (100.0%) | 0 (0.0%) | None needed |
| BMI | 2,614 (74.4%) | 899 (25.6%) | Median imputation |
| Baseline PtGADA | 2,895 (82.4%) | 618 (17.6%) | Median imputation (secondary-outcome covariate use only) |
| Baseline PPAIN | 2,892 (82.3%) | 621 (17.7%) | Median imputation (secondary-outcome covariate use only) |
| Baseline RAPID3 | 2,291 (65.2%) | 1,222 (34.8%) | Median imputation (secondary-outcome covariate use only) |

Notes: Percentages were calculated using the full analytic cohort (N=3,513) as the denominator. Missing continuous covariates were imputed using the cohort median, and missing sex values were imputed using the modal category. Diagnosis was complete and required no imputation. Baseline PtGADA, PPAIN, and RAPID3 values were imputed only when used as covariates in IPW or multivariable-adjusted sensitivity models. Outcome values were not imputed for defining outcome-specific baseline, calculating change from baseline, or estimating the primary longitudinal trajectories.

Abbreviations: BMI, body mass index; IA, inflammatory arthritis; IPW, inverse probability weighting; PPAIN, patient-reported pain assessed on a 0–100 visual analogue scale; PsA, psoriatic arthritis; PtGADA, Patient Global Assessment of Disease Activity assessed on a 0–100 visual analogue scale; RA, rheumatoid arthritis; RAPID3, Routine Assessment of Patient Index Data 3; SpA, spondyloarthritis.

**Supplementary Table S3. Proportions of patients achieving clinically meaningful improvement at predefined assessment time points by action-plan completion intensity.**

| **Outcome** | **Threshold** | **Time point** | **0 plans n (%) [95% CI]** | **1–2 plans n (%) [95% CI], p value vs 0 plans** | **≥3 plans n (%) [95% CI], p value vs 0 plans** |
| --- | --- | --- | --- | --- | --- |
| **PtGADA** | Δ ≤ −10 | W12 | 49; 19 (38.8%) [26.4–52.8%] | 87; 28 (32.2%) [23.3–42.6%], p=0.438 | 303; 151 (49.8%) [44.2–55.4%], p=0.151 |
|  | Δ ≤ −10 | W24 | 27; 5 (18.5%) [8.2–36.7%] | 41; 19 (46.3%) [32.1–61.3%], p=0.019 | 185; 80 (43.2%) [36.3–50.4%], p=0.014 |
|  | Δ ≤ −10 | W48 | 12; 0 (0.0%) [0.0–24.2%] | 17; 9 (52.9%) [31.0–73.8%], p=0.003 | 79; 36 (45.6%) [35.0–56.5%], p=0.003 |
| **PPAIN** | Δ ≤ −10 | W12 | 49; 14 (28.6%) [17.8–42.4%] | 87; 23 (26.4%) [18.3–36.6%], p=0.788 | 303; 135 (44.6%) [39.1–50.2%], p=0.036 |
|  | Δ ≤ −10 | W24 | 27; 9 (33.3%) [18.6–52.2%] | 41; 12 (29.3%) [17.6–44.5%], p=0.723 | 185; 77 (41.6%) [34.8–48.8%], p=0.413 |
|  | Δ ≤ −10 | W48 | 12; 1 (8.3%) [1.5–35.4%] | 17; 7 (41.2%) [21.6–64.0%], p=0.093 | 79; 34 (43.0%) [32.7–54.0%], p=0.025 |
| **RAPID3** | Δ ≤ −3.6 | W12 | 71; 14 (19.7%) [12.1–30.4%] | 60; 12 (20.0%) [11.8–31.8%], p=0.968 | 230; 65 (28.3%) [22.8–34.4%], p=0.153 |
|  | Δ ≤ −3.6 | W24 | 60; 8 (13.3%) [6.9–24.2%] | 26; 5 (19.2%) [8.5–37.9%], p=0.483 | 153; 64 (41.8%) [34.3–49.8%], p<0.001 |
|  | Δ ≤ −3.6 | W48 | 16; 4 (25.0%) [10.2–49.5%] | 11; 4 (36.4%) [15.2–64.6%], p=0.675 | 78; 11 (14.1%) [8.1–23.5%], p=0.277 |

Notes: Responders were defined as patients achieving at least a 10-point improvement from baseline for PtGADA and PPAIN, and at least a 3.6-point improvement for RAPID3. Follow-up observations within ±30 days of the nominal landmark were included. Percentages are calculated among participants with available outcome data at the corresponding time point. P-values are from chi-squared tests versus the zero-plans reference group. 95% CIs are Wilson score intervals.

Abbreviations: CI, confidence interval; PPAIN, patient-reported pain assessed on a 0–100 visual analogue scale; PtGADA, Patient Global Assessment of Disease Activity; RAPID3, Routine Assessment of Patient Index Data 3; VAS, visual analogue scale; W12, week 12; W24, week 24; W48, week 48.

**Supplementary Table S4. Outcome availability and follow-up by engagement group**

| **Outcome / Group** | **Baseline n** | **W12 n (%)** | **W24 n (%)** | **W48 n (%)** | **W12 rate** | **Note** |
| --- | --- | --- | --- | --- | --- | --- |
| **PtGADA** | 2,895 | 439 (15.2%) | 253 (8.7%) | 108 (3.7%) |  | Overall |
| 0 plans | 2,050 | 49 (2.4%) | 27 (1.3%) | 12 (0.6%) | 2.4% | High attrition |
| 1–2 plans | 434 | 87 (20.0%) | 41 (9.4%) | 17 (3.9%) | 20.0% | Moderate |
| ≥3 plans | 411 | 303 (73.7%) | 185 (45.0%) | 79 (19.2%) | 73.7% | High retention |
| **PPAIN** | 2,892 | 439 (15.2%) | 253 (8.7%) | 108 (3.7%) |  | Overall |
| 0 plans | 2,047 | 49 (2.4%) | 27 (1.3%) | 12 (0.6%) | 2.4% | High attrition |
| 1–2 plans | 434 | 87 (20.0%) | 41 (9.4%) | 17 (3.9%) | 20.0% | Moderate |
| ≥3 plans | 411 | 303 (73.7%) | 185 (45.0%) | 79 (19.2%) | 73.7% | High retention |
| **RAPID3** | 2,291 | 361 (15.8%) | 239 (10.4%) | 105 (4.6%) |  | Overall |
| 0 plans | 1,579 | 71 (4.5%) | 60 (3.8%) | 16 (1.0%) | 4.5% | High attrition |
| 1–2 plans | 362 | 60 (16.6%) | 26 (7.2%) | 11 (3.0%) | 16.6% | Moderate |
| ≥3 plans | 350 | 230 (65.7%) | 153 (43.7%) | 78 (22.3%) | 65.7% | High retention |

Notes: Follow-up rates were calculated as the proportion of patients with baseline data for that outcome who provided at least one valid observation within the landmark window (±30 days).

Abbreviations: PPAIN, patient-reported pain assessed on a visual analogue scale; PtGADA, Patient Global Assessment of Disease Activity; RAPID3, Routine Assessment of Patient Index Data 3; VAS, visual analogue scale; W12, week 12; W24, week 24; W48, week 48.

**Supplementary Table S5. Selected primary and prespecified sensitivity analyses**

| **Analysis** | **PtGADA** | **PPAIN** | **RAPID3** | **Interpretation** |
| --- | --- | --- | --- | --- |
| Primary model (overall slope, 0–114 weeks) | −0.050/wk  (p=0.187) | −0.017/wk  (p=0.559) | −0.001/wk  (p=0.930) | No outcome showed a statistically significant unstratified overall slope once baseline-only patients are excluded |
| Global test for group differences  (Wald χ², df=4) | χ²=22.84  p=1.4×10−4 | χ²=11.90  p=0.018 | χ²=12.11  p=0.017 | Overall engagement-group differences were statistically significant for all outcomes. |
| Group main effects only  (Wald χ², df=2) | χ²=19.37  p=6.2×10−5 | χ²=10.40  p=0.0055 | χ²=10.83  p=0.0044 | Group separation remained significant for all outcomes; the group × time interaction was not significant for any outcome (Results) |
| Early-engagement proxy  (first five AP slots; week ≥4) | ≥3 vs 0: −11.54  p<0.001 | ≥3 vs 0: −7.75  p=0.004 | Not assessed | Dependence on engagement accumulated over the full follow-up period was reduced; reverse causation and immortal-time bias could not be eliminated. |
| Alternative thresholds  (0 vs ≥1; 0 vs ≥3) | ≥3 vs 0: −9.62, p<0.001  ≥1 vs 0: −8.51, p<0.001 | ≥3 vs 0: −6.51, p=0.002  ≥1 vs 0: −5.62, p=0.006 | ≥3 vs 0: −0.80, p=0.096  ≥1 vs 0: −0.66, p=0.154 | A graded engagement-associated pattern was observed for PtGADA and PPAIN; the RAPID3 signal was weaker and not significant at either threshold. |
| Truncation at 60 weeks | −0.110/wk  (p=0.055) | −0.044/wk  (p=0.413) | +0.009/wk  (p=0.415) | Unstratified slopes remained non-significant after truncation at 60 weeks. |
| Truncation at 80 weeks | −0.078/wk  (p=0.109) | −0.014/wk  (p=0.732) | +0.010/wk  (p=0.211) | Pattern consistent with the 60-week truncation; the unstratified slope remained non-significant. |

Notes: Unless otherwise specified, estimates were derived from longitudinal linear models with patient-level cluster-robust standard errors. Negative coefficients or slopes indicate improvement. Engagement-group coefficients compare patients completing ≥3 plans with those completing 0 plans, unless another exposure threshold is explicitly stated. Wald χ² p values represent joint robust tests of the relevant engagement-group terms. The early-engagement analysis classified exposure using the first five action-plan slots and included observations from week 4 onward. Time-window analyses restricted follow-up to 60 or 80 weeks. All p values are two-sided.

Abbreviations: AP, action plan; df, degrees of freedom; PPAIN, patient-reported pain assessed on a 0–100 visual **analogue** scale; PtGADA, Patient Global Assessment of Disease Activity; RAPID3, Routine Assessment of Patient Index Data 3; VAS, visual **analogue** scale; wk, week.

**Supplementary Table S6. Recorded baseline therapy classes by action-plan completion group among patients with baseline PtGADA (N=2,895)**

| **Therapy class** | **0 plans, n (%)** | **1–2 plans, n (%)** | **≥3 plans, n (%)** | **p value (≥3 vs 0)** |
| --- | --- | --- | --- | --- |
| Any recorded medication | 544 (26.5%) | 159 (36.6%) | 194 (47.2%) | <0.001 |
| csDMARD | 203 (9.9%) | 73 (16.8%) | 77 (18.7%) | <0.001 |
| bDMARD | 166 (8.1%) | 53 (12.2%) | 45 (10.9%) | 0.059 |
| tsDMARD/JAK inhibitor | 27 (1.3%) | 15 (3.5%) | 15 (3.6%) | <0.001 |
| Glucocorticoid | 106 (5.2%) | 31 (7.1%) | 49 (11.9%) | <0.001 |
| NSAID | 117 (5.7%) | 29 (6.7%) | 44 (10.7%) | <0.001 |
| Analgesic (non-NSAID) | 39 (1.9%) | 15 (3.5%) | 18 (4.4%) | 0.002 |

Notes: Percentages were calculated within each engagement group (0 plans, n=2,050; 1–2 plans, n=434; ≥3 plans, n=411). Medication records were voluntarily self-logged in the application; absence of a recorded medication cannot be distinguished from non-logging.

Abbreviations: bDMARD, biologic disease-modifying antirheumatic drug; csDMARD, conventional synthetic disease-modifying antirheumatic drug; JAK, Janus kinase; NSAID, nonsteroidal anti-inflammatory drug; PtGADA, Patient Global Assessment of Disease Activity; tsDMARD, targeted synthetic disease-modifying antirheumatic drug.

**Supplementary Figure S1. Longitudinal Routine Assessment of Patient Index Data 3 (RAPID3) trajectories according to digital self-management engagement.** Model-derived mean change from baseline with 95% confidence intervals (CIs) and follow-up availability by engagement group are shown. Because baseline observations were not included as model rows, estimates at or near week 0 are model extrapolations rather than observed baseline changes.

**Supplementary Figure S2. Individual Patient Global Assessment of Disease Activity (PtGADA) responses at week 12 according to digital self-management engagement.** Each bar represents one participant; negative values indicate improvement. The dashed line indicates the 10-point minimal clinically important difference (MCID) threshold.
